# Impact of adjuvant chemotherapy on survival after pathological complete response in rectal cancer: a meta-analysis of 31,558 patients

**DOI:** 10.1007/s00384-024-04668-x

**Published:** 2024-06-24

**Authors:** Francisco Cezar Aquino de Moraes, Francinny Alves Kelly, Maria Eduarda Cavalcanti Souza, Rommel Mario Rodríguez Burbano

**Affiliations:** 1 Federal University of Pará, Rua Augusto Corrêa, nº 01, Guamá, Belém, Pará, 66073-000 Brazil; 2 Department of Hypertension, Dante Pazzanese Institute of Cardiology, Sao Paulo, Brazil; 3 University of Pernambuco, Recife, Pernambuco, Brazil; 4 Ophir Loyola Hospital, Belém, Pará, Brazil

**Keywords:** Rectal cancer, Adjuvant chemotherapy, Pathological complete response, Meta-analysis

## Abstract

**Background:**

Locally advanced rectal cancer (LARC) typically involves neoadjuvant chemoradiotherapy (nCRT) followed by surgery (total mesorectal excision, TME). While achieving a complete pathological response (pCR) is a strong indicator of a positive prognosis, the specific benefits of adjuvant chemotherapy after pCR remain unclear. To address this knowledge gap, we conducted a systematic review and meta-analysis to assess the potential advantages of adjuvant therapy in patients who achieve pCR.

**Methods:**

In this study, we searched Medline, Embase, and Web of Science databases for relevant research. We focused on binary outcomes, analyzing them using odds ratios (ORs) with 95% confidence intervals (CIs). To account for potential variability between studies, all endpoints were analyzed with DerSimonian and Laird random-effects models. We assessed heterogeneity using the *I*^2^ statistic and employed the R statistical software (version 4.2.3) for all analyses.

**Results:**

Thirty-four studies, comprising 31,558 patients, were included. The outcomes demonstrated a significant difference favoring the AC group in terms of overall survival (OS) (HR 0.75; 95% CI 0.60–0.94; *p* = 0.015; *I*^2^ = 0%), and OS in 5 years (OR 1.65; 95% CI 1.21–2.24; *p* = 0.001; *I*^2^ = 39%). There was no significant difference between the groups for disease-free survival (DFS) (HR 0.94; 95% CI 0.76–1.17; *p* = 0.61; *I*^2^ = 17%), DFS in 5 years (OR 1.19; 95% CI 0.82–1.74; *p* = 0.36; *I*^2^ = 43%), recurrence-free survival (RFS) (HR 1.10; 95% CI 0.87–1.40; *p* = 0.39; *I*^2^ = 0%), and relapse-free survival (OR 1.08; 95% CI 0.78–1.51; *p* = 0.62; *I*^2^ = 0%).

**Conclusion:**

This systematic review and meta-analysis found a significant difference in favor of the ACT group in terms of survival after pCR. Therefore, the administration of this treatment as adjuvant therapy should be encouraged in clinical practice.

**Supplementary Information:**

The online version contains supplementary material available at 10.1007/s00384-024-04668-x.

## Introduction

Colorectal cancer (CRC) is a leading cause of cancer morbidity and mortality worldwide [1]. Notably, younger patients with CRC often present with aggressive tumor types diagnosed at advanced stages [2, 3]. Among CRC cases, locally advanced rectal cancer (LARC) represents a significant challenge in clinical practice [4]. Currently, the standard treatment for LARC consists of neoadjuvant chemoradiotherapy (NCRT) followed by total mesorectal excision (TME) [5, 6]. Microsatellite instability-high (MSI-H) or mismatch repair-deficient (dMMR) tumors, identified by specific tests (PCR sequencing or immunohistochemistry), are present in a small subset of LARC patients (1–3%) [7, 8]. In these patients, neoadjuvant immunotherapy has shown remarkable complete clinical response (cCR) rates [7, 8]. However, the majority of LARC cases involve microsatellite stable (MSS) or mismatch repair-proficient (pMMR) tumors. For these patients, a multimodal approach combining (chemo)radioimmunotherapy holds greater promise than immunotherapy alone. While NCRT offers advantages such as improved local tumor control and sphincter preservation, its effectiveness is variable, with only a minority of patients (10–30%) achieving complete pathological response (pCR) [6, 9, 10]. This translates to a high rate of tumor recurrence (25–40%) [11].

Complete pathological response (pCR), defined as the absence of viable tumor cells in the surgical specimen after neoadjuvant chemoradiotherapy (NCRT), is a significant milestone in the treatment of locally advanced rectal cancer (LARC)[12]. This absence of tumor cells is a strong indicator of favorable prognosis, associated with a considerable improvement in survival and a reduction in the risk of disease recurrence [13, 14]. pCR is associated with an approximately 70% reduction in the risk of local tumor recurrence and a 50% decrease in the risk of death from colorectal cancer (CRC) [15–17].

Although pCR has a positive prognostic value, the need for adjuvant chemotherapy (ACT) after NCRT for patients with pCR is still not fully understood. Current treatment guidelines, such as those from the National Comprehensive Cancer Network (NCCN) [18], recommend ACT for all patients who have received NCRT, regardless of their response; however, the impact of ACT on long-term survival outcomes in patients with pCR is inconclusive, and previous studies have shown conflicting results. Thus, this systematic review and meta-analysis aims to comprehensively evaluate the impact of ACT on the treatment of LARC patients who achieve pCR after NCRT.

## Methods

### Protocol and registration

This systematic review followed the Preferred Reporting Items for Systematic Reviews and Meta-Analysis (PRISMA) guidelines [19] (PRISMA Checklist, Supplementary Tables S1 and S2). The protocol was registered in the International Prospective Register of Systematic Reviews (PROSPERO), National Institute for Health and Care Research (NIHR) on December 13th, 2023, with registration number CRD42024519913.

This meta-analysis investigated the impact of adjuvant therapy (ACT) on treatment outcomes in patients with rectal cancer. To select the studies, we used the PICO question: Population (P), patients diagnosed with rectal cancer; Intervention (I), receipt of adjuvant therapy (ACT); Comparison (C), patients who did not receive it (no-ACT); Outcomes (O), evaluation of the impact of ACT on overall survival (OS), disease-free survival (DFS), and relapse-free survival (RFS).

### Eligibility criteria

Studies that met the following eligibility criteria were included: (1) patients diagnosed with rectal cancer who received neoadjuvant chemoradiotherapy; (2) studies comparing ACT with No-ACT after pCR; (3) outcome of interest: OS, DFS, and RFS. We excluded studies with overlapping populations, single-arm clinical trials, and studies without results of interest.

We therefore sought to answer the following question: Doess(ACT) improve the outcomes of patients with rectal cancer that achieved pCR after neoadjuvant chemotherapy and subsequent surgery?

### Search strategy

On January 25, 2024, a systematic search was conducted across three databases: PubMed, Embase, and Web of Science. The search strategy employed MeSH terms, the details of which are provided in Table S4 of the Supplementary Material. In an effort to include additional studies, the references of the articles included, as well as systematic reviews of the literature, were evaluated. An alert was set up in each database to notify of any newly published studies that matched the search criteria. The studies identified in the databases and in the references of the articles were integrated into the reference management software (EndNote®, version X7, Thomson Reuters, Philadelphia, USA). Duplicate articles were excluded both automatically and manually. The titles and abstracts of the articles found in the databases were independently analyzed by two reviewers (F.C.A.d.M. and R.M.R.B).

### Data extraction and endpoints

We extracted the following information: (1) study design; (2) age; (3) gender; (3) adjuvant chemotherapy; (4) adjuvant radiotherapy; (5) country of study; (6) overall survival (OS); (7) disease-free survival (DFS); and relapse-free survival (RFS).

We define the following: overall survival (OS), time elapsed from diagnosis to death from any cause [20]; disease-free survival (DFS), time elapsed from diagnosis to first local or regional recurrence, distant metastasis, or death from any cause [21]; and relapse-free survival (RFS), time elapsed from diagnosis to first local or regional recurrence [22].

Two authors (F.A.K. and F.C.A.M.) collected baseline characteristics and pre-specified outcome data. Whenever available, we consulted the full protocol of each study to check the objectives, population, and other relevant information about the design and conduct of the study. For publications reporting results from the same study, the most recent or complete publication was considered.

### Risk of *bias* assessment

The quality assessment of observational studies was performed using the Newcastle–Ottawa Scale (NOS), in which studies are scored on a 0 to 9 scale according to selection, comparability, and exposure criteria [23]. Two authors (F.C.A.M. and R.M.R.B.) independently conducted the risk of bias assessment, and disagreements were resolved by consensus. Funnel-plot analyses were employed to examine publication bias [24].

We used the Grading of Recommendations, Assessment, Development, and Evaluations (GRADE) approach to assess the overall quality of the evidence obtained by the included RCTs [25]. This framework categorizes evidence quality into four levels based on the assessment of the methodological limitations, inconsistency, imprecision, indirectness, and publication bias: very low, low, moderate, and high. For this evaluation, we used GRADEpro GDT software (Copyright © 2020, McMaster University and Evidence Prime Inc., USA).

### Statistical analysis

Risk ratio (OR) was used to analyze the binary outcomes, with 95% confidence intervals (CI). We consider OR > 1 favoring the control (No-ACT) group and OR < 1 favoring the intervention group (ACT). The Cochrane *Q*-test and *I*^2^ statistics were used to assess heterogeneity; *p* values > 0.10 and *I*^2^ values > 25% were considered to indicate significance for heterogeneity [26]. The Sidik–Jonkman estimator was used to calculate the tau^2^ variance between studies [27]. We used DerSimonian and Laird random-effect models for all endpoints [28]. Publication bias was explored using Egger’s linear regression test [29]. Statistical analyses were performed using R statistical software, version 4.2.3 (R Foundation for Statistical Computing).

## Results

### Search results and characteristics of included studies

**Fig. 1:**
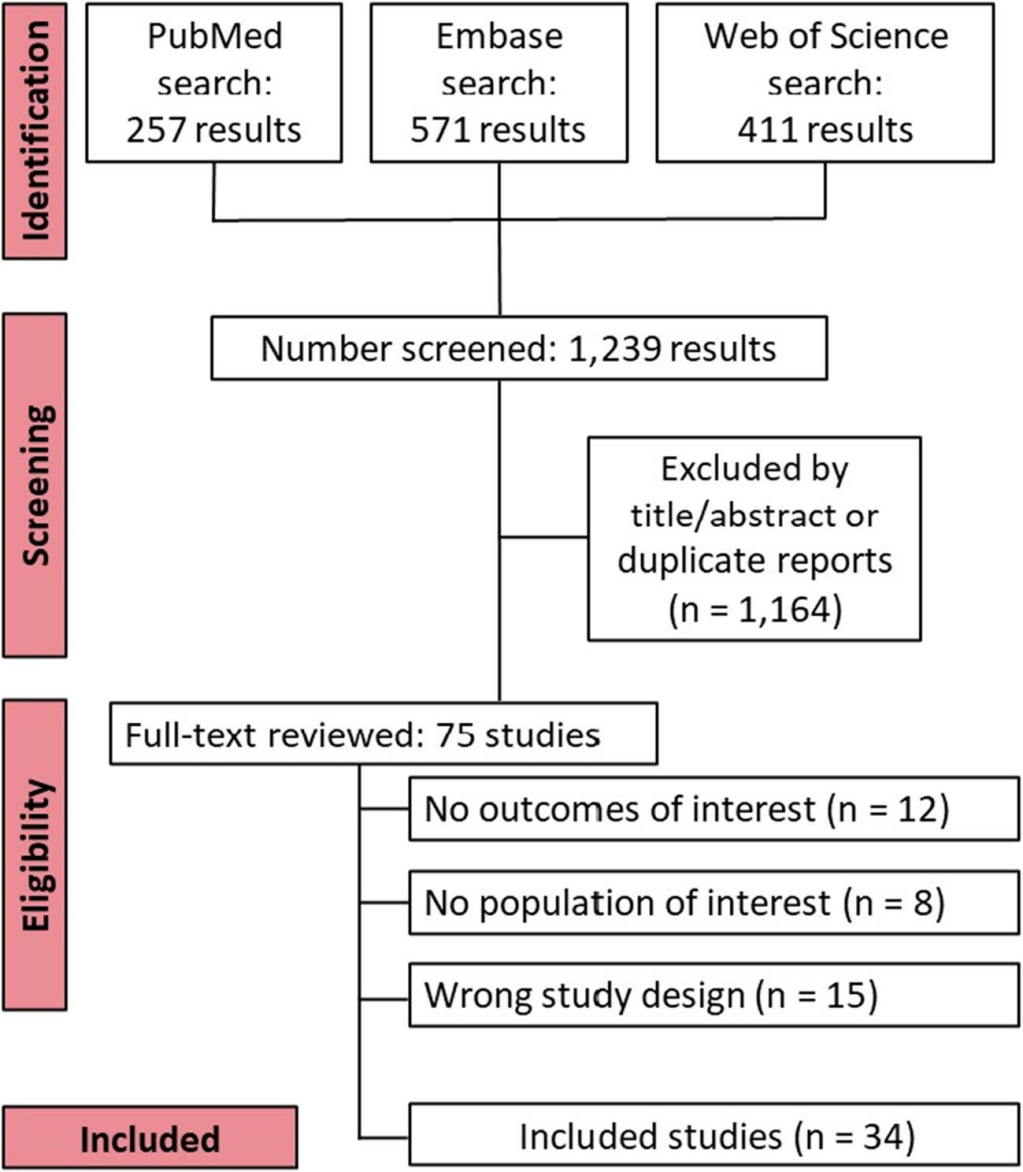
Preferred Reporting Items for Systematic Review and Meta-Analysis (PRISMA) flow diagram of study screening and selection

The selection was detailed in a PRISMA flow diagram (Fig. 1). A total of 1239 references were retrieved in our systematic search. After removing duplicate records and assessing the studies based on title and abstract, 1164 references were excluded and 75 full-text manuscripts were eligible and thoroughly reviewed for inclusion and exclusion criteria. Of these, 34 studies satisfied the eligibility criteria and formed the scope of the analysis, comprising 31,558 patients [30–63].

A total of 31,558 patients were divided into 11,804 patients in the group ACT and 19,754 in the group No-ACT. The majority of the patients were on clinical stage II (13,378) with the median age ranging from 52.9 to 65.7 years. The follow-up ranged from 35 to 120 months. Study patient baseline characteristics of the included studies are summarized in Table 1.

**Table 1:** Design and characteristics of studies included in the meta-analysis

Study, year	Coutry	Study design	No. of patients		Age, years	Chemotherapy regime	Clinical stage	Follow-up, months	NOS
			ACT	No-ACT					
Bliggenstorfer (2022)	USA	Retrospective	494	1054	Mean: 60.21	NA	II = 4524 III = 3418	NA	7
Capirci (2008)	Italy	Retrospective	127	439	Mean: 61.8	5-FU, capecitabine, raltitrexed, 5-FU + Mitomycin C, 5-FU + cisplatin, oxaliplatin + 5-FU, oxaliplatin + raltitrexed, oxaliplatin + capecitabine	II = 250 III = 254	45.6 moths	6
Chen (2023)	China	Retrospective	207	73	Median: 53.1	FOLFOX mFOLFOX6	II = 88 III = 192	55 months	8
Dossa (2018)	USA	Retrospective	667	667	Median: 56.5	NA	NA	36.9 months	8
Fukui (2022)	Japan	Retrospective	75	30	NA	5-FU, FL, FOLFOX	NA	49 months	9
Gahagan (2020)	USA	Retrospective	1513	4319	Mean: 59.83	NA	NA	NA	7
Gamaleldin (2017)	USA	Retrospective	47	83	Mean: 58.9	5-FU, FL	II = 73 III = 56	68.4 months	9
Gave (2014)	Israel	Retrospective	35	17	Median: 65.7	5-FU, capecitabine	NA	49.4 months	7
Govindarajan (2011)	USA	Retrospective	64	9	NA	5-FU-based, FL, FOLFOX	NA	69.6 months	9
He (2020)	China	Retrospective	712	297	Median: 55	Ora/i.v. fluoropyrimidine, capecitabine, FL, CapeOX, FOLFOX, FOLFOXIRI, FOLFIRI	II = 229 III = 780	35 months	9
Hu (2019)	China	Retrospective	56	115	Mean: 56.5	Capecitabine, CapeOX	II = 55 III = 116	120 months	7
Jiang (2021)	China	Retrospective	187	60	NA	Capecitabine, CapeOX	II = 67 III = 180	53 months	8
Kim (2017)	Korea	Retrospective	50	40	NA	FL, capecitabine	NA	70.7 months	7
Kiran (2012)	USA	Retrospective	14	34	NA	5-FU, FL	NA	52.6 months	6
Kuan (2016)	China	Retrospective	114	115	Mean: 59.59	FL, tegafur, capecitabine	II = 87 III = 172	37 months	7
Kuo (2022)	China	Retrospective	115	155	NA	5-FU, capecitabine, oxaliplatin, leucovorin, UFUR FL, capecitabine, FOLFOX, CapeOX, 5-FU + oxaliplatin	NA	50.88 months	7
Lai (2023)	USA	Retrospective	780	1441	Median: 60.59	NA	II = 1083 III = 1138	50.9 months	8
Lee (2015)	Korea	Retrospective	32	12	NA	Capecitabine, uracil-tegafur, doxifluridine, capecitabine	NA	60.5 months	9
Lichthardt (2017)	Germany	Retrospective	9	15	NA	5-FU, capecitabine, FOLFOX, FOLFIRI	NA	NA	5
Lorenzon, 2017	Italy	Retrospective	77	155	NA	Oral/i.v. fluoropyrimidine	NA	47.6 months	7
Lu (2018)	China	Retrospective	22	29	NA	CapeOX, capecitabine, FOLFOX, oxaliplatin + S-1	NA	50 months	7
Mass (2015)	Netherlands	Retrospective	290	608	Mean: 61	FL, FOLFOX, 5-FU,capecitabine, CapeOX	NA	NA	7
Morris (2021)	USA	Retrospective	778	1643	Median: 60.64	NA	II = 1233 III = 1188	42.3 months	8
Nafouje (2022)	USA	Retrospective	1292	1292	Mean: 57.2	NA	II = 1123 III = 1461	56.4 months	7
Nguyen (2019)	USA	Retrospective	60	36	Mean: 58.14	5-FU, capecitabine, FULFOX	II = 25 III = 71	77.76 months	8
Peng (2018)	China	Retrospective	83	22	Mean: 52.9	CapeOX	II = 35 III = 70	49 months	5
Polanco (2018)	USA	Retrospective	741	741	NA	NA	II = 698 III = 784	39 months	7
Shahab (2017)	USA	Retrospective	789	2102	Mean: 60.1	NA	II = 1612 III = 1279	NA	7
Tay (2016)	Australia	Retrospective	97	29	NA	Oral/i.v. fluoropyrimidine, capecitabine, FOLFOX, FL	NA	45.5 months	8
Turner (2018)	USA	Retrospective	1379	2726	Mean: 57.7	NA	II = 2183 III = 1922	NA	7
Voss (2020)	USA	Retrospective	139	54	NA	5-FU, capecitabine, FOLFOX, CapeOX, oxaliplatin	NA	63 months	7
Xu (2016)	USA	Retrospective	484	1243	NA	NA	NA	NA	8
Yeo (2010)	Korea	Retrospective	256	48	NA	5-FU, FL, FOLFOX, FOLFIRI, capecitabine, oral/i.v. fluoropyrimidine	NA	43 months	7
Zhou (2016)	China	Retrospective	19	21	Mean: 54.05	CapeOX, FOLFOX4, capecitabine	II = 13 III = 22	57 months	7

*USA* United States of America, *ACT* Adjuvant chemotherapy, *No* number of patients, *NA* not available, *FOLFOX* folinic acid + fuorouracil + oxaliplatin, *FU* fuorouracil, *FL* fuorouracil + leucovorin, *CapeOX* capecitabine + oxaliplatin, *FOLFIRI* folinic acid + fuorouracil + irinotecan, *NOS* Newcastle–Ottawa Scale

### Overall survival

**Fig. 2:**
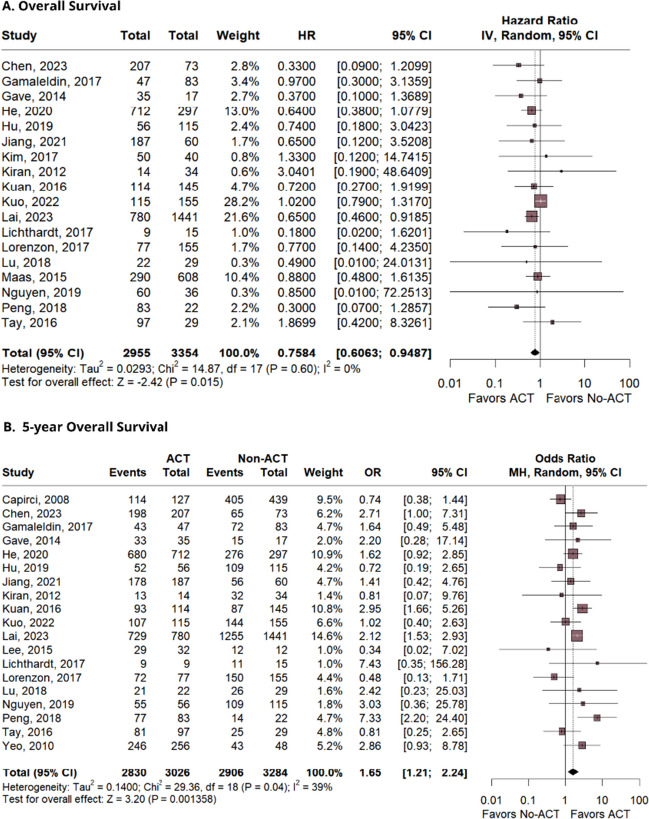
The analysis of the overall survival and 5-year overall survival

OS was significantly prolonged in patients that received ACT versus No-ACT (HR 0.75; 95% CI 0.60–0.94; *p* = 0.015; *I*^2^ = 0%; Fig. 2A). The 5-year analysis also showed a statistically improved OS for the ACT group (OR 1.65; 95% CI 1.21–2.24; *p* = 0.001; *I*^2^ = 39%; Fig. 2B).

### Disease-free survival

**Fig. 3:**
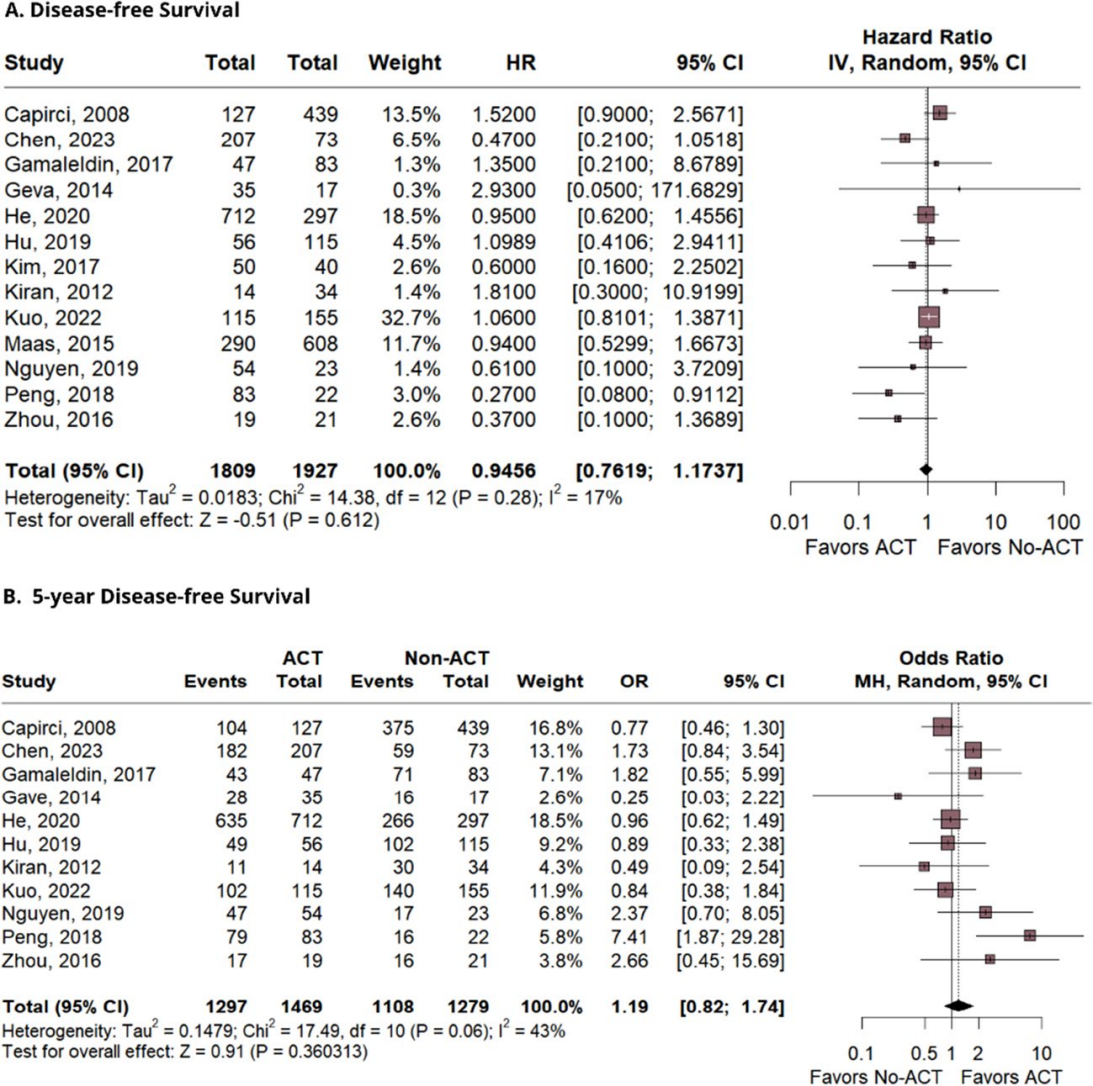
The findings for disease-free survival and 5-year disease-free survival

Analysis of DFS was based in 13 studies, which directly compared ACT versus No-ACT. A total of 1809 patients were included in the intervention group and 1927 in the control group. Figure 3A presents the following findings: (HR 0.94; 95% CI 0.76–1.17; *p* = 0.61; *I*^2^ = 17%; Fig. 3A). There was also no significant impact on DFS observed in the 5 years analysis (OR 1.19; 95% CI 0.82–1.74; *p* = 0.36; *I*^2^ = 43%; Fig. 3B).

### Recurrence-free survival

**Fig. 4:**
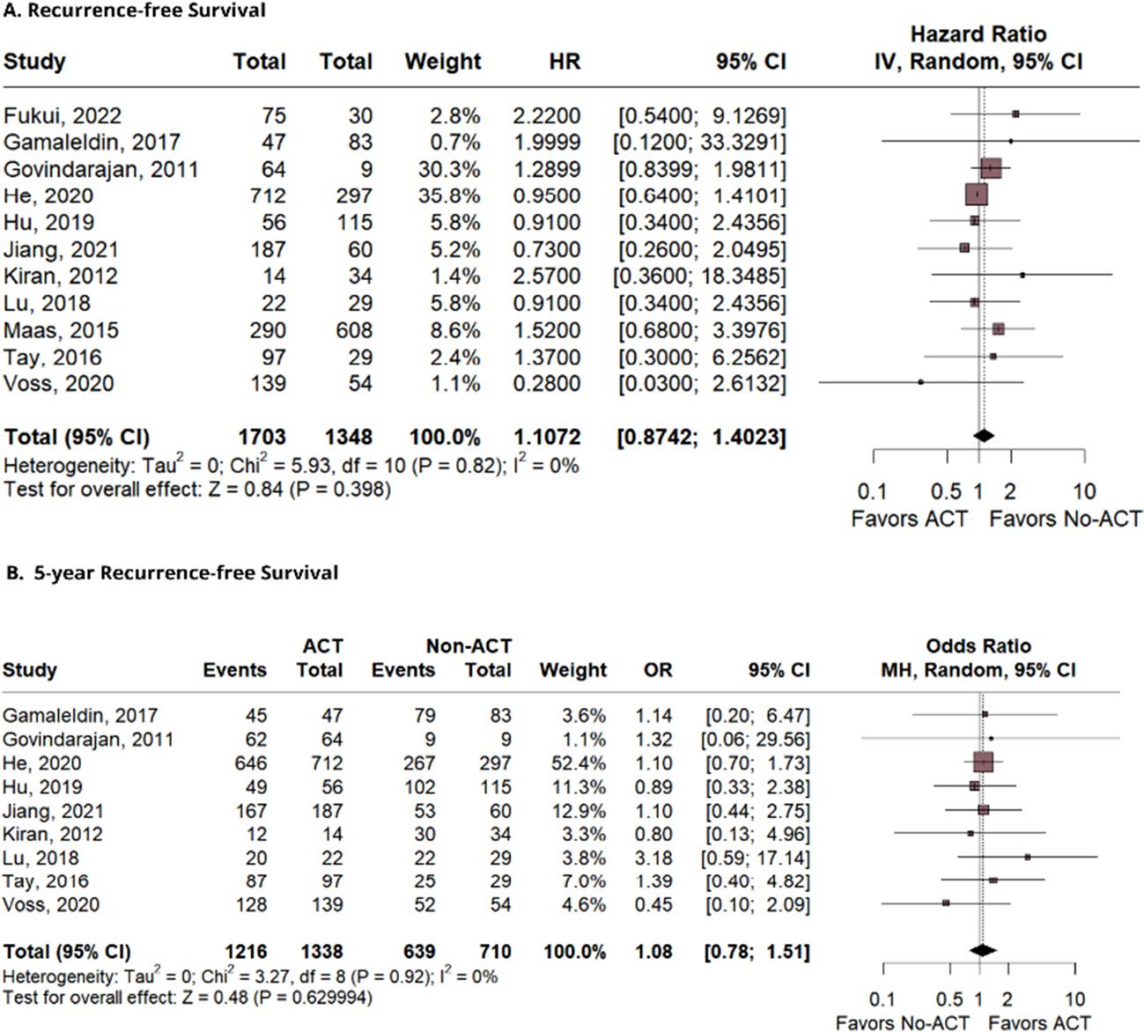
The analysis of recurrence-free survival and 5-year recurrence-free survival

RFS data were available for 11 studies; thus, 1703 and 1348 patients were included respectively in the experimental and control groups. The analysis, shown in Fig. 4A, yielded a (HR 1.10; 95% CI 0.87–1.40; *p* = 0.39; *I*^2^ = 0%). The 5-year RFS analysis showed no statistical significance (OR 1.08; 95% CI 0.78–1.51; *p* = 0.62; *I*^2^ = 0%; Fig. 4B).

### Sensitivity analysis

We performed a leave-one-out sensitivity analysis for all outcomes. In general, heterogeneity was low in the majority of the outcomes assessed in this meta-analysis (*I*^2^ < 25%). Our overall analysis showed an increased heterogeneity in the 5-year OS and 5-year DFS outcomes (*I*^2^ = 43%). Despite conducting the sensitivity analysis, we were unable to identify the study responsible for the increased heterogeneity in OS. However, for DFS, by omitting Peng et al. [46], there was a substantial decrease in the heterogeneity for this outcome. The leave-one-out sensitivity analysis of the main outcomes is detailed in Supplementary Figure S1.

### Estimation of publication *bias*

**Fig. 5:**
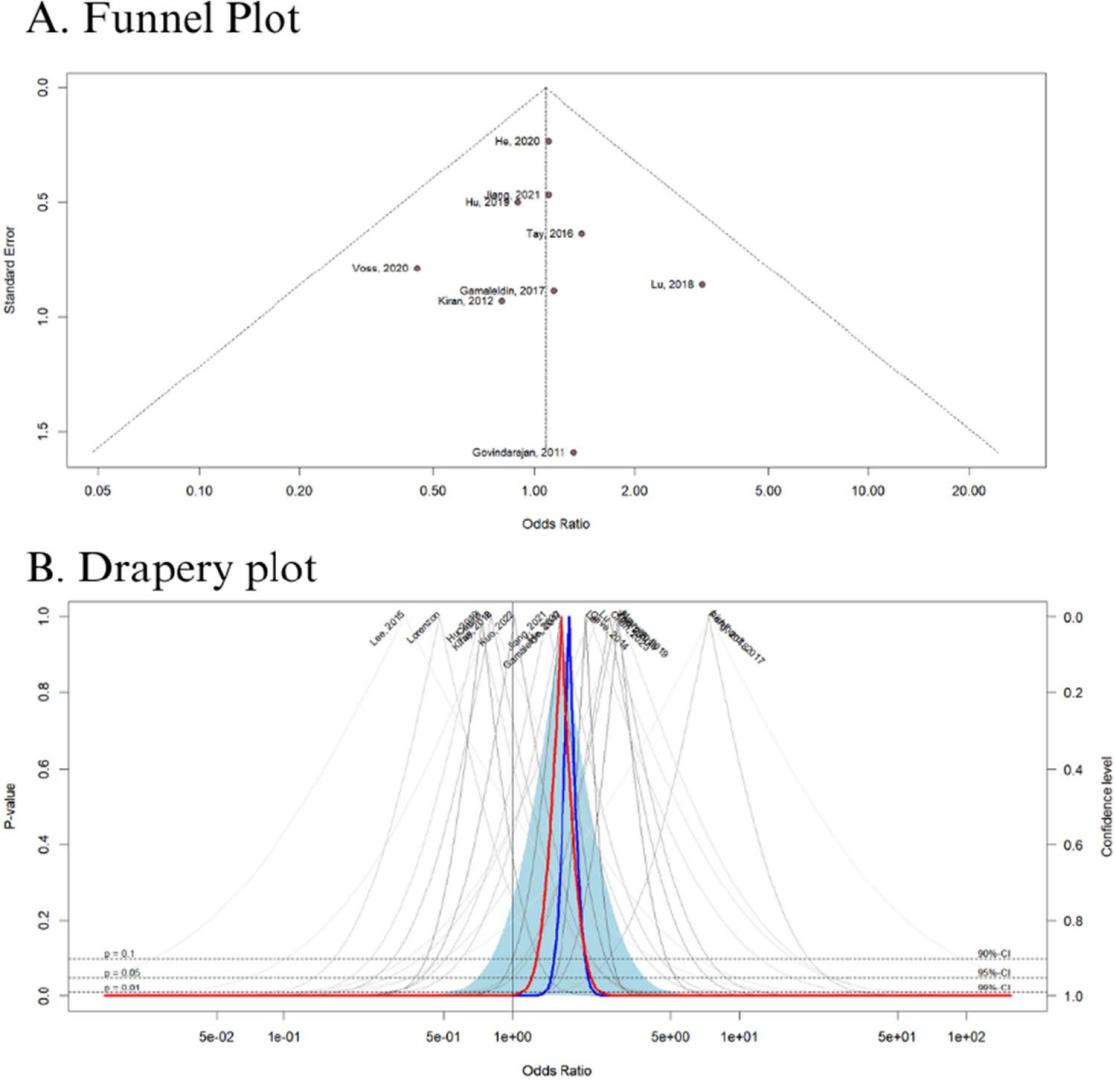
**A** Funnel plot analysis of the disease-free survival shows no evidence of publication bias. **B** Drapery plot showing curve with significant *p*-value

We conducted a funnel plot analysis for all outcomes. The *X*-axis corresponds to the odds ratio, while the *Y*-axis represents the standard error. The dashed lines indicate two standard errors on either side of the mean effect. Each circle is representative of one study. Test for asymmetry was statistically significant by Begg’s rank correlation between precision and effect size, and Egger’s regression test. In Fig. 5A, the symmetrical distribution of comparable studies in the funnel plot indicates that there is no evidence of publication bias in the outcomes comparing ACT versus No-ACT (Fig. 5A). Furthermore, the drapey plot result confirms the high reliability of our results (*p* = 0.01) (Fig. 5B). The funnel plot analysis of the main outcomes is detailed in Supplementary Figure S2.

### Quality assessment

The individual assessment of each observational study included in the meta-analysis is depicted in Figure S3. All studies scored between 5 and 9 points. Overall, 13 of 34 studies were deemed at good quality. The studies by Peng et al. and Lichthardt et al. scored 5 points and were of poor quality due to not fulfilling the minimum criteria for outcome follow-up domains.

According to the GRADE assessment, the 5-year OS combined data from our 19 observational studies, the OS analysis combined 18 studies, while DFS was based on 13 studies, 5-year DFS and RFS on 11, and 5-year RFS was based on 9 studies. The GRADE quality assessment is detailed in the Supplementary Fig. 4.

## Discussion

This systematic review and meta-analysis investigated the impact of adjuvant chemotherapy (ACT) versus no treatment (No-ACT) on overall survival (OS), disease-free survival (DFS), and recurrence-free survival (RFS) in patients with locally advanced rectal cancer after achieving complete pathological response (pCR) to neoadjuvant treatment. Our results demonstrate a substantial benefit for ACT in OS (HR 0.75; 95% CI 0.60–0.94; *p* = 0.015), indicating its ability to prolong survival compared to the control group. These findings are particularly encouraging, suggesting ACT as a potential therapeutic strategy for this patient group.

CR is a heterogeneous tumor type with a variety of possible treatments [64]. Most can be treated with surgery alone; however, a significant proportion of patients present with LARC require NAT with the aim of reducing tumor burden and increasing the effectiveness of the surgical procedure [65]. Understanding the clinical outcomes after pCR would help guide the precise selection of patients who would benefit from this intervention and drive the personalization of cancer treatment.

However, we observed no significant effect of ACT on DFS (HR 0.94; 95% CI 0.76–1.17; *p* = 0.61) or RFS (HR 1.10; 95% CI 0.87–1.40; *p* = 0.39). These results might imply that ACT may not directly prevent initial recurrence in patients with rectal cancer.

Several factors can explain the discrepancy between DFS and RFS with OS. ACT may act to eradicate micrometastases and residual disease after initial neoadjuvant treatment, contributing to the revitalization of the immune system and the control of molecular and biochemical mechanisms associated with initial disease progression [30, 66]. Thus, it is possible that these combined effects justify the benefit of this treatment for increased survival, even without preventing initial disease recurrence in treated patients.

Additionally, the inconclusive impact of ACT on DFS and RFS may be partially explained by the limitations of traditional follow-up methods. Studies have shown that some patients with early-stage disease experience delayed recurrences, which can only be captured through longer monitoring periods [12]. This highlights the need for extended follow-up, a need further emphasized by the emergence of circulating tumor DNA (ctDNA) as a promising tool.

Current methods for recurrence assessment in LARC, such as colonoscopy and carcinoembryonic antigen (CEA), have significant limitations. Colonoscopy, while offering high sensitivity and specificity for colorectal cancer diagnosis, can be met with patient resistance due to its invasive nature and potential complications [67–69]. CEA, the only biomarker recommended by the National Comprehensive Cancer Network for postoperative surveillance, suffers from insufficient sensitivity and specificity, limiting its effectiveness [70–72]. As a marker for residual micrometastases, ctDNA has recently demonstrated its ability to identify colon cancer patients who benefit from ACT based on postoperative ctDNA levels [73, 74]. While evidence in LARC remains preliminary, ctDNA holds promise for improved risk stratification and management in this patient population as well. Positive ctDNA status has been linked to a higher risk of recurrence after colorectal surgery [75–78]. This suggests that ctDNA-positive patients with LARC might benefit from intensified postoperative ACT regimens and more frequent follow-up to ensure timely detection and treatment of potential recurrences.

While adjuvant therapy remains to be fundamental in the treatment of CRC, recent research explores the potential of tumor-infiltrating lymphocytes (TILs) and the Immunoscore for not only predicting prognosis but also potentially informing treatment decisions beyond standard therapy [79]. The presence and density of TILs within the tumor microenvironment have been linked to patient survival in CRC. The meta-analyses have shown a significant association between high TILs and improved clinical outcomes such as OS, DFS, and cancer-specific survival (CSS) [80, 81]. This suggests that a robust anti-tumor immune response, as indicated by TIL infiltration, can positively influence patient prognosis. The immunoscore, a standardized approach that quantifies TIL density and distribution, has also been correlated with improved prognosis in CRC patients [79, 81]. Unlike adjuvant therapy, which directly targets cancer cells, TILs and immunoscore offer a prognostic tool. By assessing the pre-existing immune response within the tumor, these approaches can help predict a patient’s risk of recurrence after standard treatment. Similar to ctDNA analysis, high TILs and immunoscore could potentially guide decisions about follow-up intensity, ensuring which LARC patients would benefit from the use of ACT following NACT and surgery.

This meta-analysis has some limitations. First, this analysis is limited by the absence of well-designed, prospective randomized controlled trials evaluating the necessity of ACT for patients with rectal cancer achieving a pCR following neoadjuvant therapy and surgery. The included studies in this analysis were exclusively retrospective cohort studies, characterized by varying sample sizes, baseline characteristics, and treatment protocols. Consequently, the presence of information bias and confounding factors was unavoidable. Second, we were unable to perform a multivariate analysis and evaluate the effect of ACT in subgroups of patients with different disease stages, individual characteristics, and specific populations, which may limit the generalizability of our results. Third, the follow-up time was variable, potentially hindering the detection of significant differences in DFS and RFS. However, despite these limitations, robust results were obtained, indicating that ACT treatment has a potential benefit for OS. The low heterogeneity found in all outcomes: OS (*I*^2^ = 0%), DFS (*I*^2^ = 17%), and RFS (*I*^2^ = 0%), reinforces the reliability of the results found in this study. Future prospective long-term studies are needed to confirm and validate our findings.

## Conclusion

In conclusion, this meta-analysis provides compelling evidence that adjuvant chemotherapy (ACT) improves overall survival in rectal cancer patients after complete pathological response, by uncertain mechanisms that are nor explained by improved disease specif survival or decreased cancer recurrence. Although ACT did not show a significant impact on disease-free survival and recurrence-free survival, the benefit in overall survival justifies the consideration of this therapeutic approach as part of the clinical management of these patients. Further research is needed to identify potential biomarkers and determine which patients would benefit from the use of adjvant chemotherapy following neoadjuvant and surgery.

## Supplementary Information

Below is the link to the electronic supplementary material.

Supplementary file1 (DOCX 222 KB)

## Data Availability

All data generated and/or analyzed during this study are included in this published article (and its supplementary information files).

## Abbreviations

CSS: Cancer-specific survival
CEA: Carcinoembryonic antigen
ctDNA: Circulating tumor DNA
CRC: Colorectal cancer
LARC: Locally advanced rectal cancer
NAT: Neoadjuvant chemoradiotherapy
TME: Total mesorectal resection
pCR: Pathological complete response
NCCN: National Comprehensive Cancer Network
ACT: Adjuvant chemotherapy
OS: Overall survival
DFS: Disease-free survival
RFS: Recurrence-free survival
CRC: Colorectal cancer
NOS: Newcastle–Ottawa Scale
HR: Hazard ratio
OR: Odds ratio
CI: Confidence interval
TILS: Tumor-infiltrating lymphocytes
TNT: Total neoadjuvant therapy
PRISMA: Preferred Reporting Items for Systematic Review and Meta-Analysis
PROSPERO: International Prospective Register of Systematic Reviews
USA: United States of America
