# Supplementary material for: Impact of adjuvant chemotherapy on survival after pathological complete response in rectal cancer: a meta-analysis of 31,558 patients
Source: Int J Colorectal Dis. 2024 Jun 24;39(1):96. doi: 10.1007/s00384-024-04668-x (PMC11196358; doi:10.1007/s00384-024-04668-x)
Supplement: Supplementary file 1 — Supplementary file1 (DOCX 222 KB) [file 384_2024_4668_MOESM1_ESM.docx]

**Supplementary Material**

**Supplementary Table 1** PRISMA 2020 Checklist.

**Supplementary Table 2** PRISMA 2020 for Abstract Checklist.

**Supplementary Table 3** Search strategies.

**Supplementary Figure 1** Leave-one-out sensitivity analyses. **A.** 5-year Disease-free survival. **B.** 5-year Overall survival. **C.** 5-year Recurrence-free survival.

**Supplementary Figure 2** Funnel plot. **A.** Funnel plot of 5-year Disease-free survival. **B.** Funnel plot of 5-year Overall survival. **C.** Funnel plot of 5-year Recurrence-free survival.

**Supplementary Figure 3** Methodological quality summary using NOS.

**Supplementary** **Figure 4** GRADE quality assessment summary.

**Table S1. PRISMA 2020 Checklist.**

| Section and Topic | Item # | Checklist item | | Location where item is reported |
| --- | --- | --- | --- | --- |
| TITLE | | | |  |
| Title | 1 | Identify the report as a systematic review. | | Page 1 |
| ABSTRACT | | | |  |
| Abstract | 2 | See the PRISMA 2020 for Abstracts checklist. | | Supplementary Materials: Table S2. |
| INTRODUCTION | | | |  |
| Rationale | 3 | Describe the rationale for the review in the context of existing knowledge. | | Page 3 |
| Objectives | 4 | Provide an explicit statement of the objective(s) or question(s) the review addresses. | | Page 3 |
| METHODS | | | |  |
| Eligibility criteria | 5 | Specify the inclusion and exclusion criteria for the review and how studies were grouped for the syntheses. | | Pages 3-4 |
| Information sources | 6 | Specify all databases, registers, websites, organisations, reference lists and other sources searched or consulted to identify studies. Specify the date when each source was last searched or consulted. | | Page 4 |
| Search strategy | 7 | Present the full search strategies for all databases, registers and websites, including any filters and limits used. | | Supplementary Materials: Table S3 Search Strategies |
| Selection process | 8 | Specify the methods used to decide whether a study met the inclusion criteria of the review, including how many reviewers screened each record and each report retrieved, whether they worked independently, and if applicable, details of automation tools used in the process. | | Page 3-4 |
| Data collection process | 9 | Specify the methods used to collect data from reports, including how many reviewers collected data from each report, whether they worked independently, any processes for obtaining or confirming data from study investigators, and if applicable, details of automation tools used in the process. | | Page 4 |
| Data items | 10a | List and define all outcomes for which data were sought. Specify whether all results that were compatible with each outcome domain in each study were sought (e.g. for all measures, time points, analyses), and if not, the methods used to decide which results to collect. | | Pages 4 |
|  | 10b | List and define all other variables for which data were sought (e.g. participant and intervention characteristics, funding sources). Describe any assumptions made about any missing or unclear information. | | Page NO |
| Study risk of bias assessment | 11 | Specify the methods used to assess risk of bias in the included studies, including details of the tool(s) used, how many reviewers assessed each study and whether they worked independently, and if applicable, details of automation tools used in the process. | | Page 4 |
| Effect measures | 12 | Specify for each outcome the effect measure(s) (e.g. risk ratio, mean difference) used in the synthesis or presentation of results. | | Page 4 |
| Synthesis methods | 13a | Describe the processes used to decide which studies were eligible for each synthesis (e.g. tabulating the study intervention characteristics and comparing against the planned groups for each synthesis (item #5)). | | Pages 3 and 4 |
|  | 13b | Describe any methods required to prepare the data for presentation or synthesis, such as handling of missing summary statistics, or data conversions. | | Pages 4-5 |
|  | 13c | Describe any methods used to tabulate or visually display results of individual studies and syntheses. | | Page 5 |
|  | 13d | Describe any methods used to synthesize results and provide a rationale for the choice(s). If meta-analysis was performed, describe the model(s), method(s) to identify the presence and extent of statistical heterogeneity, and software package(s) used. | | Page 5 |
|  | 13e | Describe any methods used to explore possible causes of heterogeneity among study results (e.g. subgroup analysis, meta-regression). | | Pages 5 |
|  | 13f | Describe any sensitivity analyses conducted to assess robustness of the synthesized results. | | Pages 5 |
| Reporting bias assessment | 14 | Describe any methods used to assess risk of bias due to missing results in a synthesis (arising from reporting biases). | | Pages 4 |
| Certainty assessment | 15 | Describe any methods used to assess certainty (or confidence) in the body of evidence for an outcome. | | Pages 5 |
| RESULTS | | | |  |
| Study selection | 16a | Describe the results of the search and selection process, from the number of records identified in the search to the number of studies included in the review, ideally using a flow diagram. | | Page 5  Figure 1 |
|  | 16b | Cite studies that might appear to meet the inclusion criteria, but which were excluded, and explain why they were excluded. | Page NO | |
| Study characteristics | 17 | Cite each included study and present its characteristics. | | Pages 5-7  Table 1 |
| Risk of bias in studies | 18 | Present assessments of risk of bias for each included study. | | Supplementary Materials: Figure 3. |
| Results of individual studies | 19 | For all outcomes, present, for each study: (a) summary statistics for each group (where appropriate) and (b) an effect estimate and its precision (e.g. confidence/credible interval), ideally using structured tables or plots. | | Pages 7-10  Figure 2 and 3 |
| Results of syntheses | 20a | For each synthesis, briefly summarise the characteristics and risk of bias among contributing studies. | | Pages 7-10  Supplementary Materials: Figure 4 |
|  | 20b | Present results of all statistical syntheses conducted. If meta-analysis was done, present for each the summary estimate and its precision (e.g. confidence/credible interval) and measures of statistical heterogeneity. If comparing groups, describe the direction of the effect. | | Pages 7-10  Figure2, 3, and 4 |
|  | 20c | Present results of all investigations of possible causes of heterogeneity among study results. | | Page 10  Figure S1 |
|  | 20d | Present results of all sensitivity analyses conducted to assess the robustness of the synthesized results. | | Page 10  Supplementary Materials: Figure S1 |
| Reporting biases | 21 | Present assessments of risk of bias due to missing results (arising from reporting biases) for each synthesis assessed. | | Page 10-11  Supplementary Materials: Figure S3 |
| Certainty of evidence | 22 | Present assessments of certainty (or confidence) in the body of evidence for each outcome assessed. | | Page 10-11 |
| DISCUSSION | | | |  |
| Discussion | 23a | Provide a general interpretation of the results in the context of other evidence. | | Pages 12 |
|  | 23b | Discuss any limitations of the evidence included in the review. | | Page 12 |
|  | 23c | Discuss any limitations of the review processes used. | | Page 12 |
|  | 23d | Discuss implications of the results for practice, policy, and future research. | | Page 12-13 |
| OTHER INFORMATION | | | |  |
| Registration and protocol | 24a | Provide registration information for the review, including register name and registration number, or state that the review was not registered. | | Page 2 |
|  | 24b | Indicate where the review protocol can be accessed, or state that a protocol was not prepared. | | Page 2 |
|  | 24c | Describe and explain any amendments to information provided at registration or in the protocol. | | Page 2 |
| Support | 25 | Describe sources of financial or non-financial support for the review, and the role of the funders or sponsors in the review. | | Page 14 |
| Competing interests | 26 | Declare any competing interests of review authors. | | Page 13-14 |
| Availability of data, code and other materials | 27 | Report which of the following are publicly available and where they can be found: template data collection forms; data extracted from included studies; data used for all analyses; analytic code; any other materials used in the review. | | Page 13-14 |

**Table S2. PRISMA 2020 for Abstract Checklist.**

| Section and Topic | Item # | Checklist item | Reported (Yes/No) |
| --- | --- | --- | --- |
| TITLE | | |  |
| Title | 1 | Identify the report as a systematic review. | Yes |
| BACKGROUND | | |  |
| Objectives | 2 | Provide an explicit statement of the main objective(s) or question(s) the review addresses. | Yes |
| METHODS | | |  |
| Eligibility criteria | 3 | Specify the inclusion and exclusion criteria for the review. | No |
| Information sources | 4 | Specify the information sources (e.g. databases, registers) used to identify studies and the date when each was last searched. | Yes |
| Risk of bias | 5 | Specify the methods used to assess risk of bias in the included studies. | No |
| Synthesis of results | 6 | Specify the methods used to present and synthesise results. | No |
| RESULTS | | |  |
| Included studies | 7 | Give the total number of included studies and participants and summarise relevant characteristics of studies. | Yes |
| Synthesis of results | 8 | Present results for main outcomes, preferably indicating the number of included studies and participants for each. If meta-analysis was done, report the summary estimate and confidence/credible interval. If comparing groups, indicate the direction of the effect (i.e. which group is favoured). | Yes |
| DISCUSSION | | |  |
| Limitations of evidence | 9 | Provide a brief summary of the limitations of the evidence included in the review (e.g. study risk of bias, inconsistency and imprecision). | No |
| Interpretation | 10 | Provide a general interpretation of the results and important implications. | Yes |
| OTHER | | |  |
| Funding | 11 | Specify the primary source of funding for the review. | No |
| Registration | 12 | Provide the register name and registration number. | No |

**Table S3** Search Strategies

| **Database** | **Search Strategy** |
| --- | --- |
| **PubMed** | (“rectal cancer” OR “rectal tumor” OR “rectal neoplasm”) AND (“neoadjuvant radiotherapy” OR “neoadjuvant chemoradiation” OR “neoadjuvant chemoradiotherapy” OR “neoadjuvant treatment” OR “neoadjuvant therapy” OR “preoperative radiotherapy” OR “preoperative chemoradiation” OR “preoperative chemoradiotherapy” OR “preoperative treatment” OR “preoperative therapy”) AND (“adjuvant chemotherapy” OR “adjuvant therapy” OR “adjuvant treatment” OR “postoperative chemotherapy” OR “postoperative therapy” OR “postoperative treatment”) AND (“pathological complete response” OR “complete pathological response” OR pCR OR “pathological complete regression”) |
| **Embase** | (TITLE-ABS-KEY(“rectal cancer”) OR TITLE-ABS-KEY(“rectal tumor”) OR TITLE-ABS-KEY(“rectal neoplasm”)) AND (TITLE-ABS-KEY(“neoadjuvant radiotherapy”) OR TITLE-ABS-KEY(“neoadjuvant chemoradiation”) OR TITLE-ABS-KEY(“neoadjuvant chemoradiotherapy”) OR TITLE-ABS-KEY(“neoadjuvant treatment”) OR TITLE-ABS-KEY(“neoadjuvant therapy”) OR TITLE-ABS-KEY(“preoperative radiotherapy”) OR TITLE-ABS-KEY(“preoperative chemoradiation”) OR TITLE-ABS-KEY(“preoperative chemoradiotherapy”) OR TITLE-ABS-KEY(“preoperative treatment”) OR TITLE-ABS-KEY(“preoperative therapy”)) AND (TITLE-ABS-KEY(“adjuvant chemotherapy”) OR TITLE-ABS-KEY(“adjuvant therapy”) OR TITLE-ABS-KEY(“adjuvant treatment”) OR TITLE-ABS-KEY(“postoperative chemotherapy”) OR TITLE-ABS-KEY(“postoperative therapy”) OR TITLE-ABS-KEY(“postoperative treatment”)) AND (TITLE-ABS-KEY(“pathological complete response”) OR TITLE-ABS-KEY(“complete pathological response”) OR TITLE-ABS-KEY(pCR) OR TITLE-ABS-KEY(“pathological complete regression”)) |
| **Web of Science** | (TS=(“rectal cancer”) OR TS=(“rectal tumor”) OR TS=(“rectal neoplasm”)) AND (TS=(“neoadjuvant radiotherapy”) OR TS=(“neoadjuvant chemoradiation”) OR TS=(“neoadjuvant chemoradiotherapy”) OR TS=(“neoadjuvant treatment”) OR TS=(“neoadjuvant therapy”) OR TS=(“preoperative radiotherapy”) OR TS=(“preoperative chemoradiation”) OR TS=(“preoperative chemoradiotherapy”) OR TS=(“preoperative treatment”) OR TS=(“preoperative therapy”)) AND (TS=(“adjuvant chemotherapy”) OR TS=(“adjuvant therapy”) OR TS=(“adjuvant treatment”) OR TS=(“postoperative chemotherapy”) OR TS=(“postoperative therapy”) OR TS=(“postoperative treatment”)) AND (TS=(“pathological complete response”) OR TS=(“complete pathological response”) OR TS=(pCR) OR TS=(“pathological complete regression”)) |

**Supplementary Figure 1** Leave-one-out sensitivity analyses. **A.** 5-year Disease-free survival. **B.** 5-year Overall survival. **C.** 5-year Recurrence-free survival.

**A.** 5-year Disease-free survival.


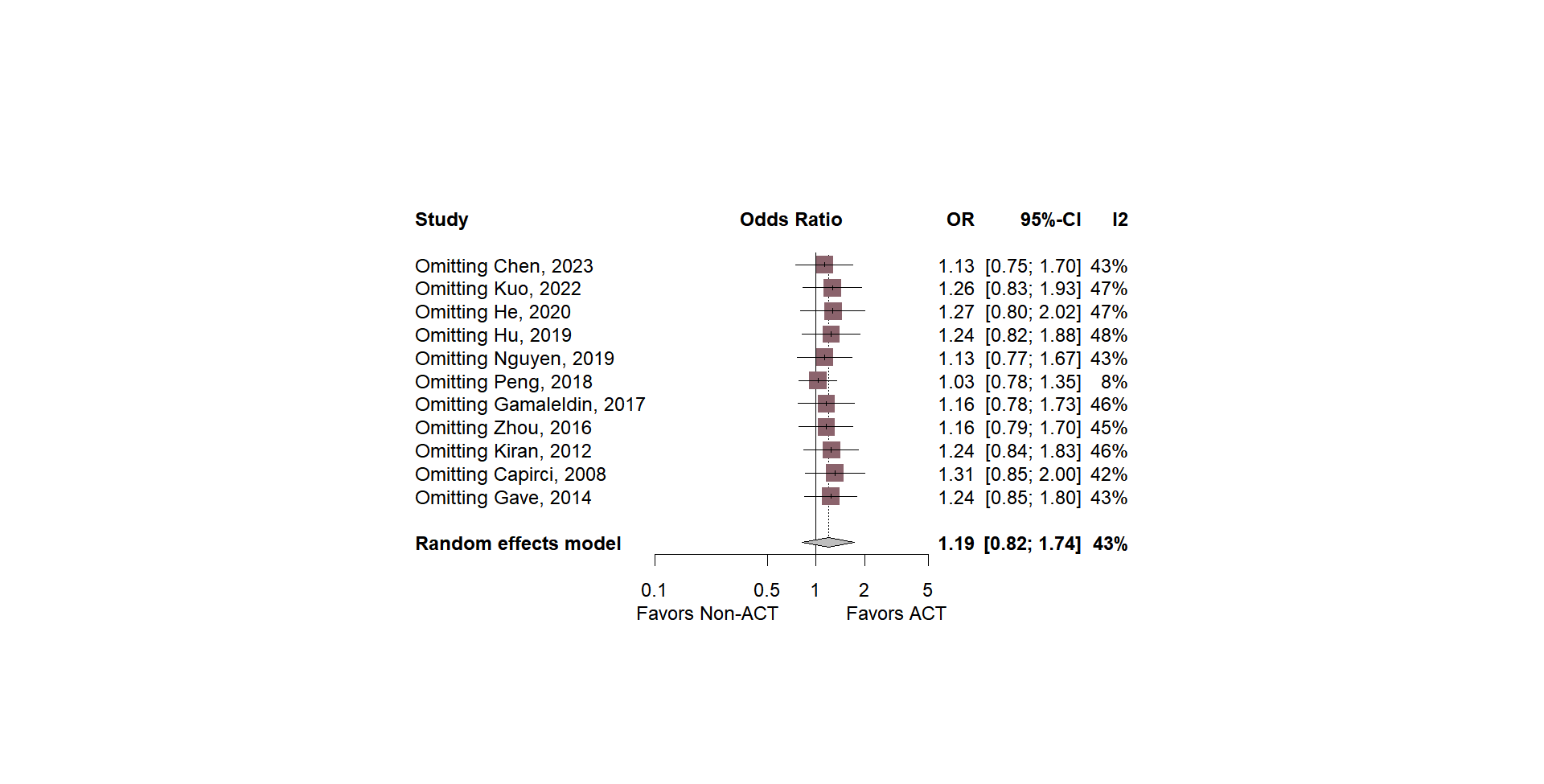


**B.** 5-year Overall survival.


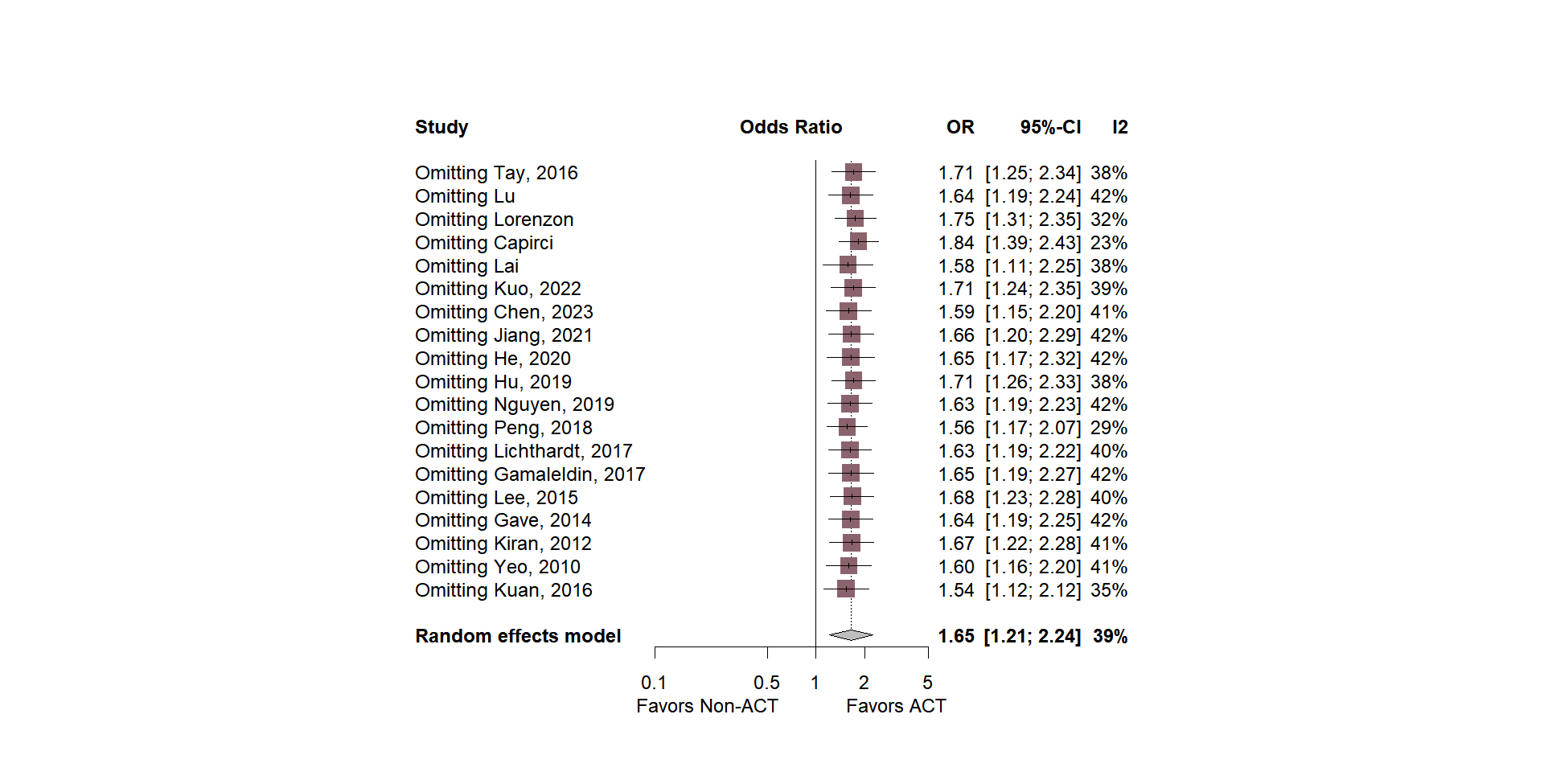


**C.** 5-year Recurrence-free survival.

**
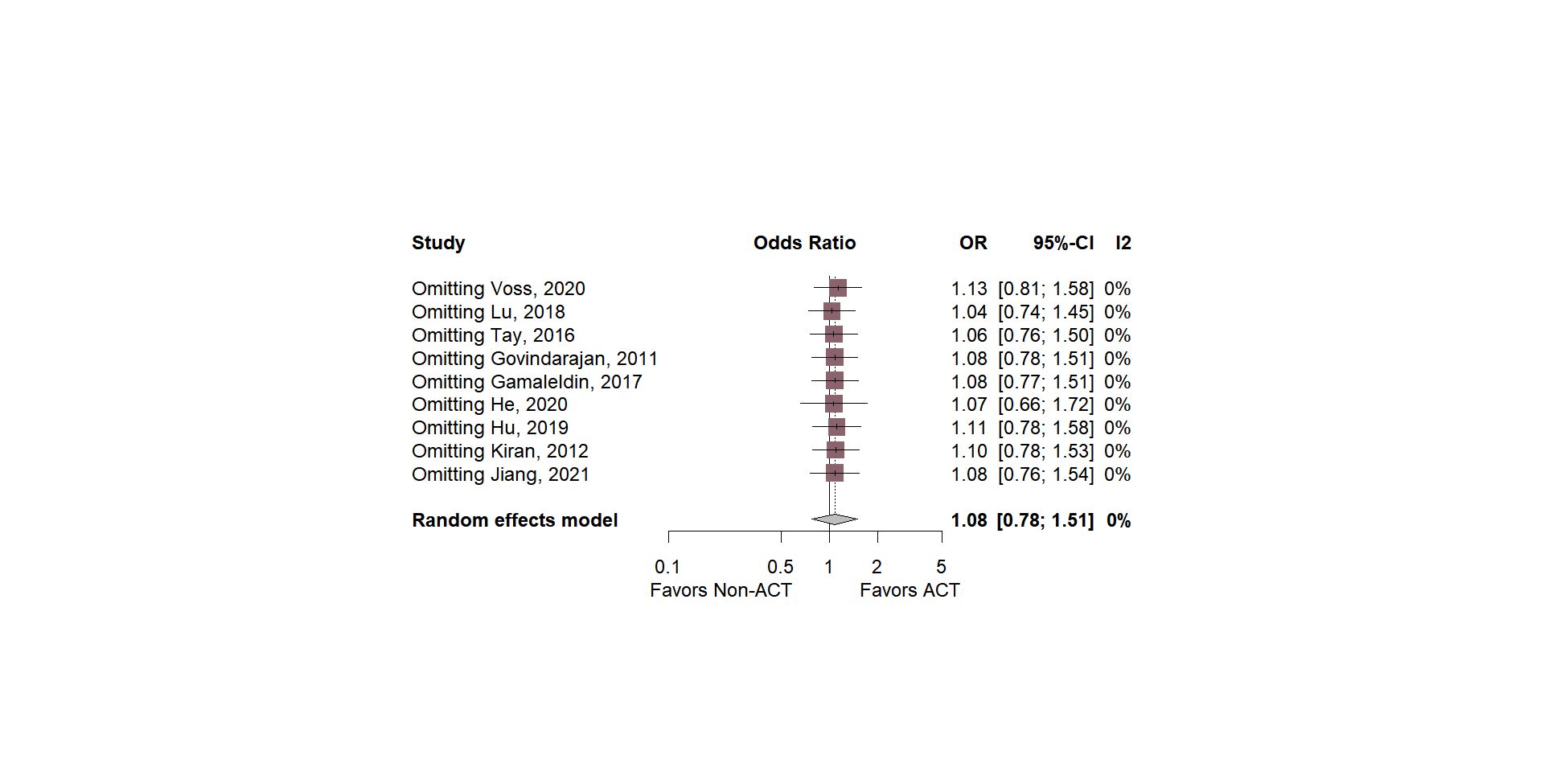
**

**Supplementary Figure 2** Funnel plot. **A.** Funnel plot of 5-year Disease-free survival. **B.** Funnel plot of 5-year Overall survival. **C.** Funnel plot of 5-year Recurrence-free survival.

**A.** Funnel plot of 5-year Disease-free survival.


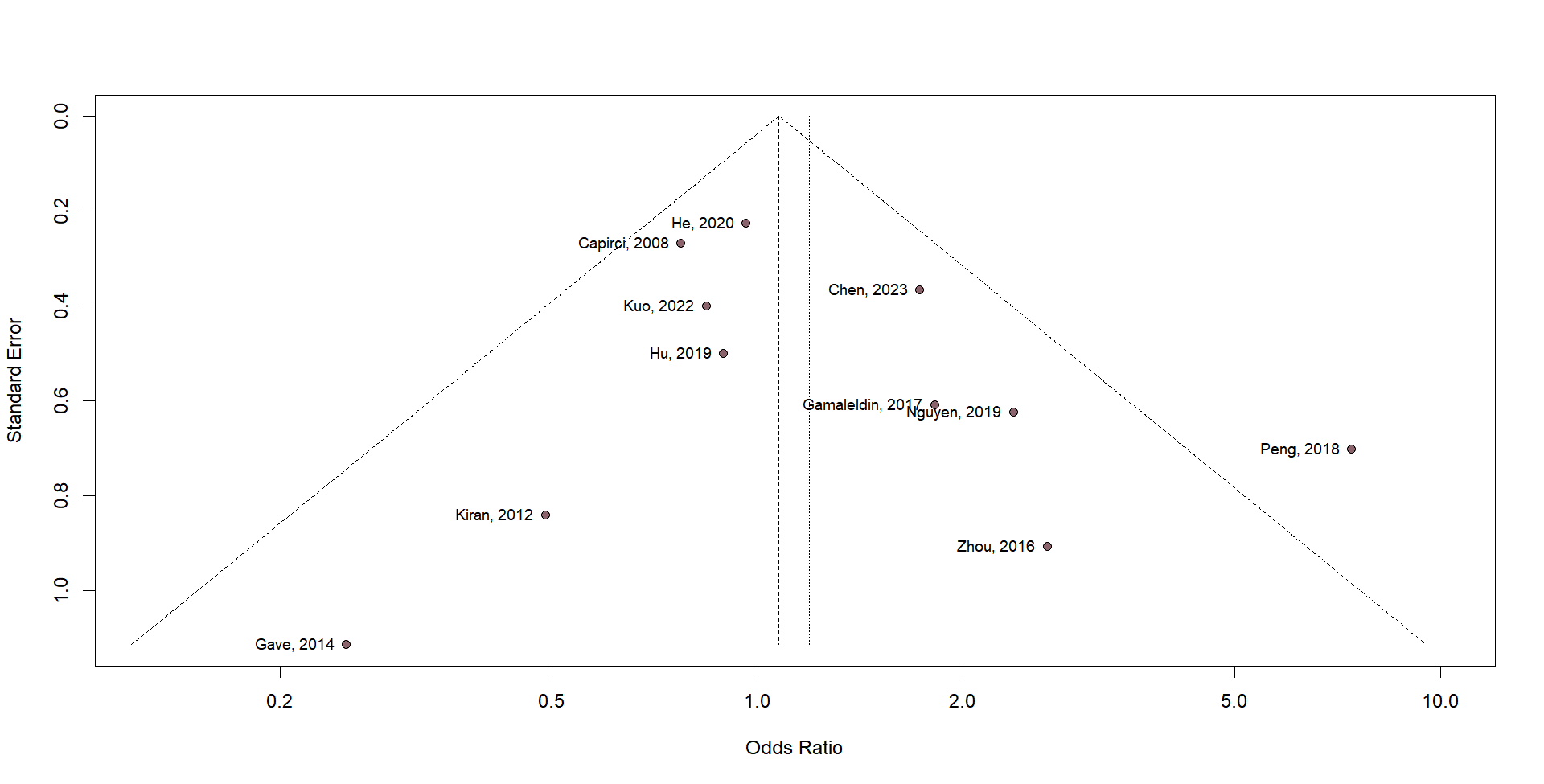


**B.** Funnel plot of 5-year Overall survival.


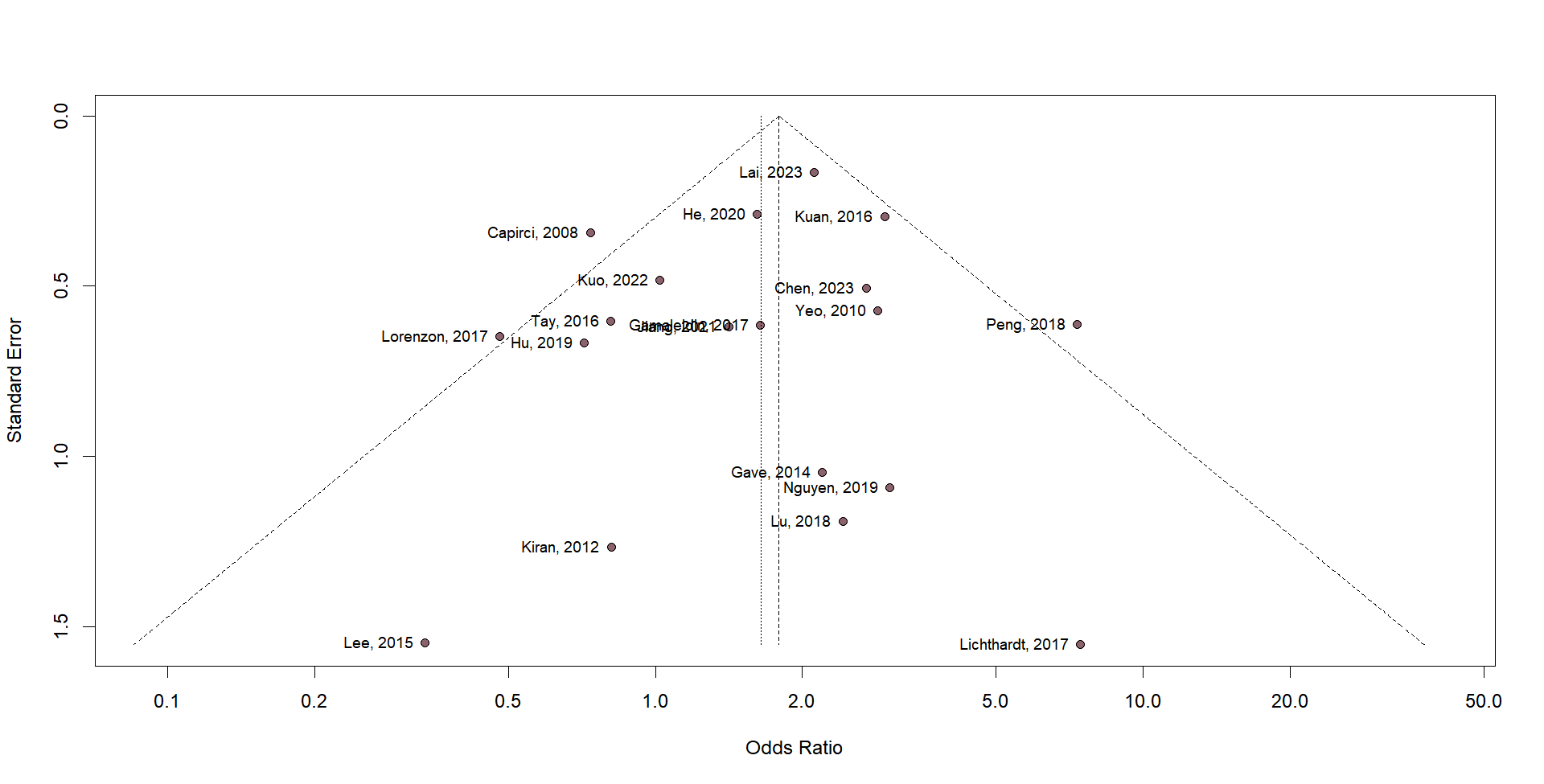


**C.** Funnel plot of 5-year Recurrence-free survival.


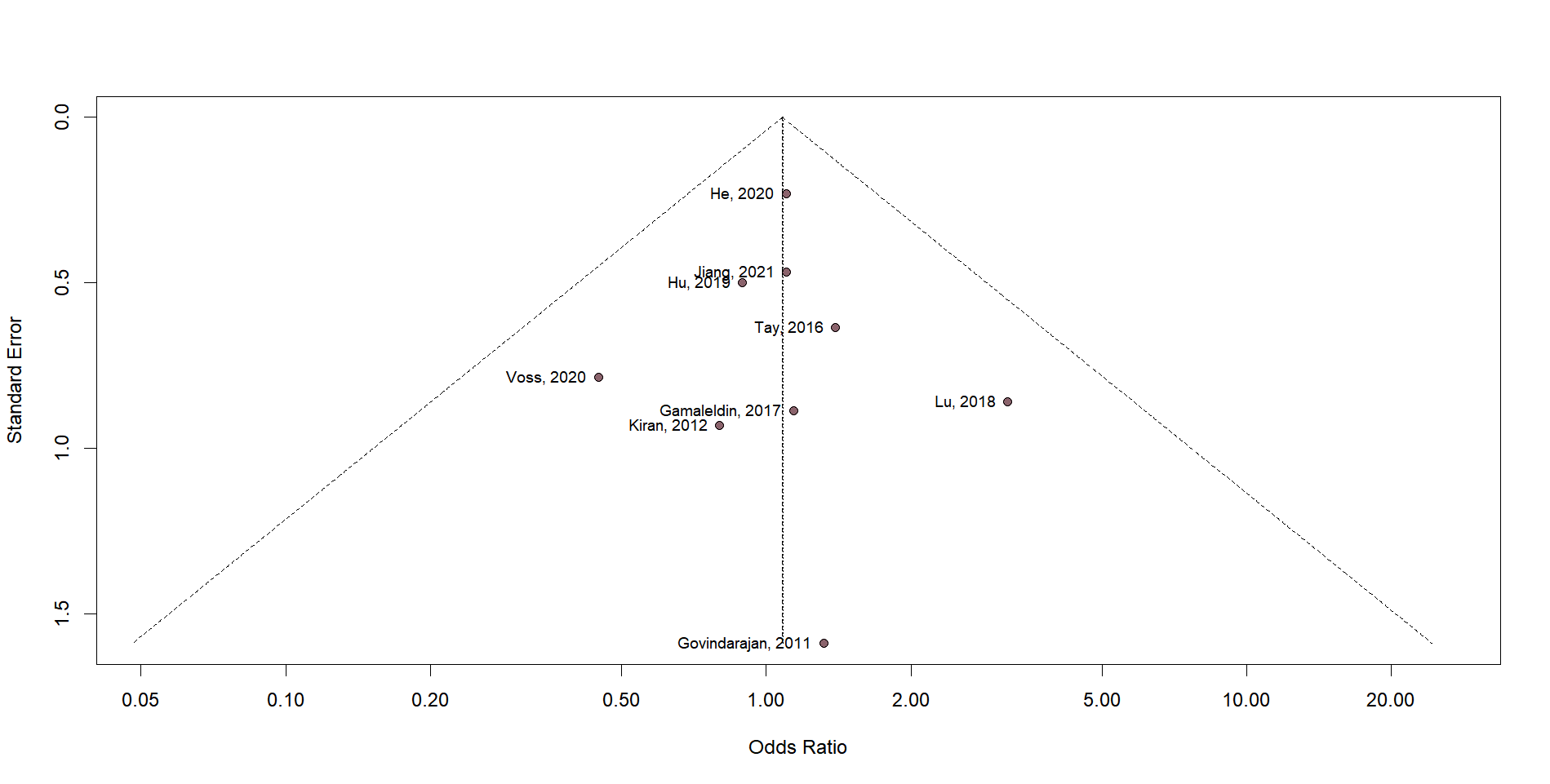


**Supplementary Figure 3** Methodological quality summary using RoB2. **A** Risk of bias domains. **B** Overall risk of bias

| **Author** | **Selection** | | | | **Comparability** | | **Outcome** | | | **Total (9/9)** |
| --- | --- | --- | --- | --- | --- | --- | --- | --- | --- | --- |
|  | **Representative of the exposed cohort** | **Selection of external control** | **Ascertainment of exposure** | **Outcome of interest not present at the start of the study** | **Main factor** | **Additional factor** | **Assessment of outcomes** | **Sufficient follow-up timea** | **Adequacy of follow-up** |  |
| Chen et al. | * | * | * | * | * | * | 0 | * | * | 8* |
| Lai et al. | 0 | * | * | * | * | * | * | * | * | 8* |
| Bliggenstorfer et al. | * | * | * | * | * | 0 | 0 | * | * | 7* |
| Fukui et al. | * | * | * | * | * | * | * | * | * | 9* |
| Kuo et al. | 0 | * | * | * | * | * | 0 | * | * | 7* |
| Nafouje et al. | 0 | * | * | * | * | * | 0 | * | * | 7* |
| Jiang et al. | * | * | * | * | * | * | 0 | * | * | 8* |
| Morris et al. | 0 | * | * | * | * | * | * | * | * | 8* |
| Gahagan et al. | * | * | * | * | * | 0 | 0 | * | * | 7* |
| He et al. | * | * | * | * | * | * | * | * | * | 9* |
| Voss et al. | 0 | * | * | * | * | * | 0 | * | * | 7* |
| Hu et al. | 0 | * | * | * | * | * | 0 | * | * | 7* |
| Nguyen et al. | * | * | * | * | * | * | 0 | * | * | 8* |
| Dossa et al. | 0 | * | * | * | * | * | * | * | * | 8* |
| Lu et al. | * | * | * | * | * | 0 | 0 | * | * | 7* |
| Peng et al. | 0 | * | 0 | * | * | 0 | 0 | * | * | 5* |
| Polanco et al. | 0 | * | * | * | * | * | 0 | * | * | 7* |
| Turner et al. | 0 | * | * | * | * | * | 0 | * | * | 7* |
| Gamaleldin et al. | * | * | * | * | * | * | * | * | * | 9* |
| Lichthardt et al. | 0 | * | 0 | * | * | 0 | 0 | * | * | 5* |
| Lorenzon et al. | 0 | * | * | * | * | * | 0 | * | * | 7* |
| Shahab et al. | 0 | * | * | * | * | * | 0 | * | * | 7* |
| Kim et al. | 0 | * | * | * | * | * | 0 | * | * | 7* |
| Kuan et al. | 0 | * | * | * | * | * | 0 | * | * | 7* |
| Tay et al. | * | * | * | * | * | * | 0 | * | * | 8* |
| Xu et al. | 0 | * | * | * | * | * | * | * | * | 8* |
| Zhou et al. | * | * | * | * | * | 0 | 0 | * | * | 7* |
| Lee et al. | * | * | * | * | * | * | * | * | * | 9* |
| Mass et al. | 0 | * | * | * | * | * | 0 | * | * | 7* |
| Gave et al. | 0 | * | * | * | * | * | 0 | * | * | 7* |
| Kiran et al. | * | * | * | * | * | 0 | 0 | 0 | * | 6* |
| Govindarajan et al. | * | * | * | * | * | * | * | * | * | 9* |
| Yeo et al. | 0 | * | * | * | * | * | 0 | * | * | 7* |
| Capirci et al. | 0 | 0 | * | * | * | * | 0 | * | * | 6* |

**Supplementary** **Figure 4** GRADE quality assessment summary.

| **Outcomes** | **Number of participants (studies)** | **Certainty of the evidence (GRADE)** | **Relative effect (95% CI)** |  |
| --- | --- | --- | --- | --- |
|  |  |  |  |  |
| Overall Survival (OS) assessed with: Hazard ratio | 12112 (18 studies) | ⨁⨁⨁◯ Moderatea | **HR 0.75 (0.60 to 0.94) [OS]** |  |
| 5-year Overall Survival (5-year OS) assessed with: Odds ratio | 12112 (19 studies) | ⨁⨁⨁◯ Moderatea | **OR 1.65 (1.21 to 2.24) [5-year OS]** |  |
| Disease-free survival (DFS) assessed with: Hazard ratio | 14168 (13 studies) | ⨁⨁⨁◯ Moderateb | **HR 0.94 (0.76 to 1.17) [DFS]** |  |
| 5-year Disease-free survival (5-year DFS) assessed with: Odds ratio | 14168 (11 studies) | ⨁⨁⨁◯ Moderateb | **OR 1.19 (0.82 to 1.74) [5-year DFS]** |  |
| Recurrence-free survival (RFS) assessed with: Hazard ratio | 12918 (11 studies) | ⨁⨁⨁◯ Moderatec | **HR 1.10 (0.87 to 1.40) [RFS]** |  |
| 5-year Recurrence-free survival (RFS) assessed with: Odds ratio | 12918 (9 studies) | ⨁⨁⨁◯ Moderatec | **OR 1.08 (0.78 to 1.51) [5-year RFS]** |  |

***The risk in the intervention group** (and its 95% confidence interval) is based on the assumed risk in the comparison group and the **relative effect** of the intervention (and its 95% CI).
**CI:** confidence interval; **HR:** hazard ratio; **OR:** odds ratio
